# “Lymphocyte population in peripheral blood in children and adolescents with graves disease. Potential predictive tool for severity of the disease”

**DOI:** 10.1007/s12020-022-03088-7

**Published:** 2022-06-02

**Authors:** Gerdi Tuli, Jessica Munarin, Beatrice Mazzucco, Patrizia Matarazzo, Luisa de Sanctis

**Affiliations:** 1 Department of Public Health and Pediatrics, University of Turin, Turin, Italy; 2 Department of Pediatric Endocrinology, Regina Margherita Children’s Hospital, Turin, Italy

**Keywords:** Graves Disease, Pediatric age, Outcome, Lymphocyte population, Predictive

## Abstract

**Purpose:**

To date, few data are available on the prognostic role of lymphocyte subsets in pediatric Graves’ Disease (GD). The aim of this retrospective study is to analyze the role of lymphocyte subtypes in predicting the severity of GD.

**Methods:**

Data of 10 pediatric subjects aged <18 years with GD onset in the period November 2017–April 2021 were collected. The lymphocyte population was assessed at the onset of GD as well as hormonal and clinical data. The follow-up period was 2.4 ± 0.8 years.

**Results:**

Pearson correlation coefficient between CD4+ /CD8+ ratio and fT3 levels and thyroid volume at diagnosis was 0.72 *(p* = *0.04)* and 0.81 *(p* = *0.004)* respectively; that between CD4+ /CD8+ ratio and the TRAb titer at diagnosis and after 6, 12 and 24 months was 0.89, 0.89, 0.73 and 0.77 respectively (*p* = *0.02*, *p* = *0.01*, *p* = *0.03* and *0.04*). The correlation coefficient of anti-thyroid drug (ATD) dose after 6 and 12 months with CD4+ /CD8 ratio was 0.88 and 0.78 (*p* = *0.001 and p* = *0.02* respectively). Patients with a higher CD4+ /CD8+ ratio at diagnosis displayed higher fT3 levels (28.73 ± 2.18 vs 13.48 ± 2.19 pmol/L, *p* = *0.03*) and higher TRAb titers (28.9 ± 11.2 vs 4.88 ± 0.97, *p* = *0.01*).

**Conclusion:**

CD4+/CD8+ ratio appears as a promising predictive tool to be considered together with other prognostic factors to better manage pediatric GD. These preliminary data need to be confirmed over a longer follow-up period and in larger cohorts.

## Introduction

Graves-Basedow disease (GD) is the most common cause of thyrotoxicosis in pediatric age even if it is considered a rare disease with an incidence of 0.3–14/100.000. The male-to-female ratio is 1:4 with the exception of the GD subtype with high IgG4 levels which appears to be more frequent in males [1]. The management of GD in pediatric age is still challenging and treatment options include medical treatment with anti-thyroid drugs (ATD), surgical treatment with total or near-total thyroidectomy, and metabolic treatment with radioiodine [2–8]. Younger age, larger goiter size, higher fT4 and fT3 levels, higher TRAb titration as well as duration of ATD treatment itself are believed to be important predictive factors [9–13]. In particular, long-term ATD treatment appears to be associated with up to 50% higher remission rates, although this is still debatable.

GD is considered an autoimmune disorder and to date the reasons for the activation of autoimmunity are still unkown but it is believed that multifactorial pathogenesis underlies GD, including polygenic susceptibility, environment and pre-existing diseases [14]. It is a humoral autoimmune disorder due to the production of TSH-R stimulating antibodies which lead to thyroid hyperfunction, increased vascularity and hyperplasia. Lymphocyte infiltration is usually present with local inflammation activated by T cells leading to cytokine release and B cells dysregulation and subsequent release of antibodies [15–18]. Prior to ATD treatment, an increase in T CD4+ and B cells and a decrease of T CD8+ cells is observed in peripheral blood [16]. After ATD treatment, a decrease in T CD4+ and B cells is observed, while the population of T CD8+ cells increases. In thyroid tissue, the CD4+ T cell subtype count is higher than in subjects without GD but lower when compared to peripheral blood of subjects with GD. The same trend is also observed for the CD8+ T cell subtype, while B lymphocytes in the thyroid tissue were present in a higher percentage than in the blood. No correlation has been noted between TRAb levels and the percentage of lymphocyte subgroups in thyroid tissue.

The aim of this prospective study is to analyze peripheral blood lymphocyte subtypes in pediatric patients with newly diagnosed GD and to investigate the potential predictive role in disease severity.

## Materials and methods

From the electronic medical records, the data of all patients aged <18 years referred to the Department of Pediatric Endocrinology of Regina Margherita Children’s Hospital for onset of GD in the period November 2017–April 2021 were collected. The Ethics Committee of the City of Health and Science University Hospital of Turin approved the study (Chairperson: M. Maddalena, Practice No.: CS2/743, date of approval: 17/11/2018). Informed consent was obtained by all parents of subjects included in the study. The diagnostic criteria for GD included: (1) elevated serum levels of thyroid hormones fT4 and fT3; (2) suppressed serum TSH levels; (3) positive serum TRAb titer. Patients with thyrotoxicosis due to hashitoxycosis or Plummer adenoma were excluded.

The complete thyroid hormone profile (TSH, fT4, fT3) and the TRAb titre were assessed at the onset of the disease and then every six months in the first 2 years. TPOAb and TGAb titres were also evaluated at the onset of the disease. Laboratory analyses were performed by Liaison^©^ Analyzer family with in vitro assay for the quantitative determination. The lymphocyte population was assessed at the onset of GD. The ultrasound data included the presence of goitre, the echogenic pattern, the vascularization of the gland and the presence of nodules. The volume of the thyroid gland was calculated using the formula: volume = 0.5 × (length × anterior-posterior depth × transverse width) and the results were expressed in SDS (individual thyroid volume × mean thyroid volume for age/SD) according to the reference values provided by Vitti et al. [19].

Pubertal development according to Tanner stages, the presence of other autoimmune diseases or other co-morbidities unrelated to GD, and familiarity for thyroid disease have also been reported. All subjects were treated with methimazole at 0.2–0.5 mg/kg/day as initial treatment and 0.1–0.2 mg/kg/day as a maintenance dosage.

Statistical analysis was performed using Graphpad 7 software (GraphPad Software, La Jolla, CA, USA) with ANOVA test to compare means between groups and Pearson’s coefficient for correlation between variables. Post-hoc tests after ANOVA were performed by Scheffe, LSD and Duncan Bonferroni tests.

## Results

The data from the 10 subjects (2 males, 8 females; age 11.7 ± 3.47 years old) with newly diagnosed GD over a follow-up period of 2.4 ± 0.8 years are presented in Table 1.

**Table 1:** Clinical features of pediatric subjects affected by Graves Disease at onset and in the first 2 years of follow-up

	At diagnosis	At 6 months	At 12 months	At 24 months
TSH mUI/l (0.6–4.84)	0.005 ± 0.001	2.46 ± 0.51	2.8 ± 0.9	2.64 ± 0.66
FT4 pmol/L (8.6–14.9)	55.09 ± 4.7	15.96 ± 5.06	21 ± 1.69	16.18 ± 4.29
FT3 pmol/L (1.87–3.99)	22.58 ± 2.87	6.72 ± 2.27	11.45 ± 5.22	7.61 ± 2.36
TRAb UI/L (<3.5)	18.25 ± 3.49	7.1 ± 2.7	17.7 ± 6.1	10.9 ± 5.8
Thyroid volume SDS	2.98 ± 0.47	—	—	—
Total lymphocyte count 10^9^/L (2.2–3.5)	2.65 ± 0.7	—	—	—
T cells 10^9^/L (1.5–2.5)	1.93 ± 0.6	—	—	—
CD4+ 10^9^/L (0.64–1.2)	1.06 ± 0.11	—	—	—
CD8+ 10^9^/L (0.64–0.9)	0.65 ± 0.1	—	—	—
CD4+ /CD8+ ratio (1.2–1.7)	2.19 ± 0.9	—	—	—
B cells 10^9^/L (0.44–0.77)	0.38 ± 0.09	—	—	—
ATD dose (mg/kg/day)	0.33 ± 0.1	0.12 ± 0.1	0.23 ± 0.1	0.09 ± 0.001

Five subjects were pre-pubertal, while the remaining five were in puberty, with different Tanner stages. All subjects showed suppressed TSH levels (0.005 ± 0.001 mUI/L) at diagnosis with high levels of fT4 (55.09 ± 4.7 pmol/L) and fT3 (22.58 ± 2.87 pmol/L). The titre of TRAb was higher at diagnosis and at 12 months (18.25 ± 3.49 and 17.7 ± 6.1 UI/L) and displayed lower levels at 6 and 24 months (7.1 ± 2.7 and 10.9 ± 5.8, *p* = *0.001*). The dose of ATD at diagnosis and at 12 months (0.33 ± 0.1 and 0.23 ± 0.1 mg/kg/day) with respect of the doses at 6 and 24 months (0.12 ± 0.1 and 0.09 ± 0.01 mg/kg/day, *p* < *0.001*) displayed the same trend. Mean thyroid volume was 2.98 ± 0.47 SDS (Table 1).

**Fig. 1:**
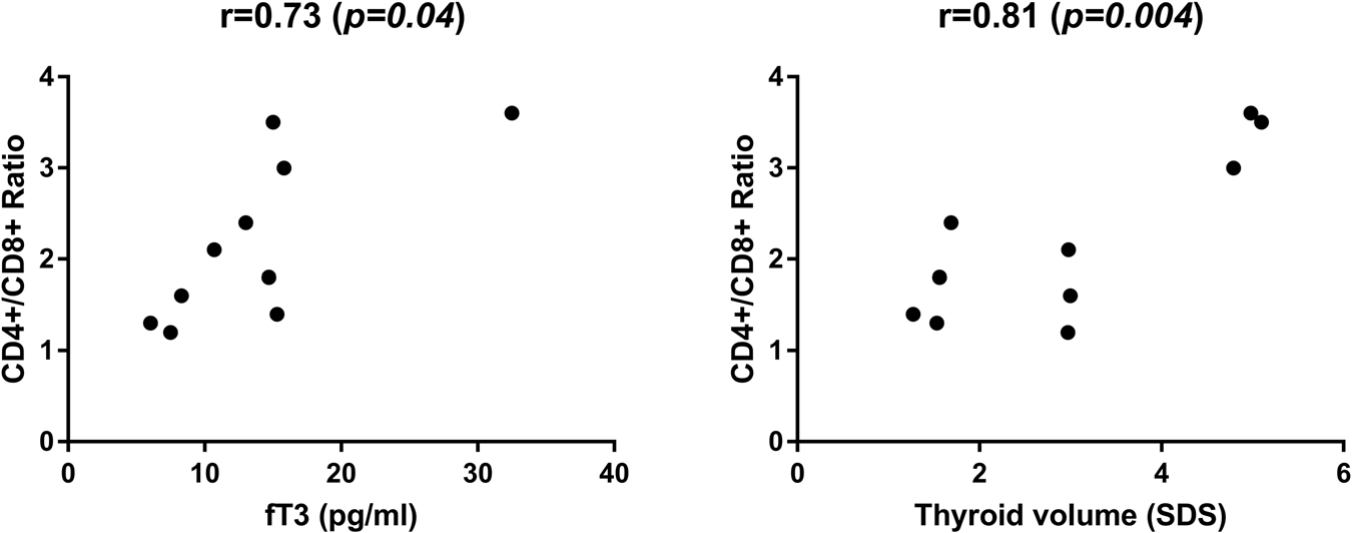
Correlation of fT3 levels and thyroid volume with CD4+/CD8+ ratio

**Fig. 2:**
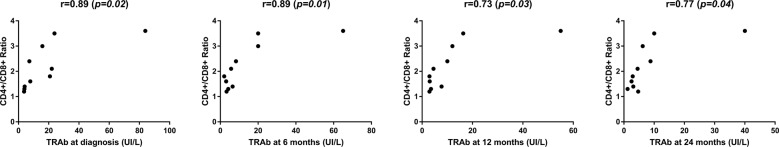
Correlation among TRAB levels and CD4+/CD8+ ratio

**Fig. 3:**
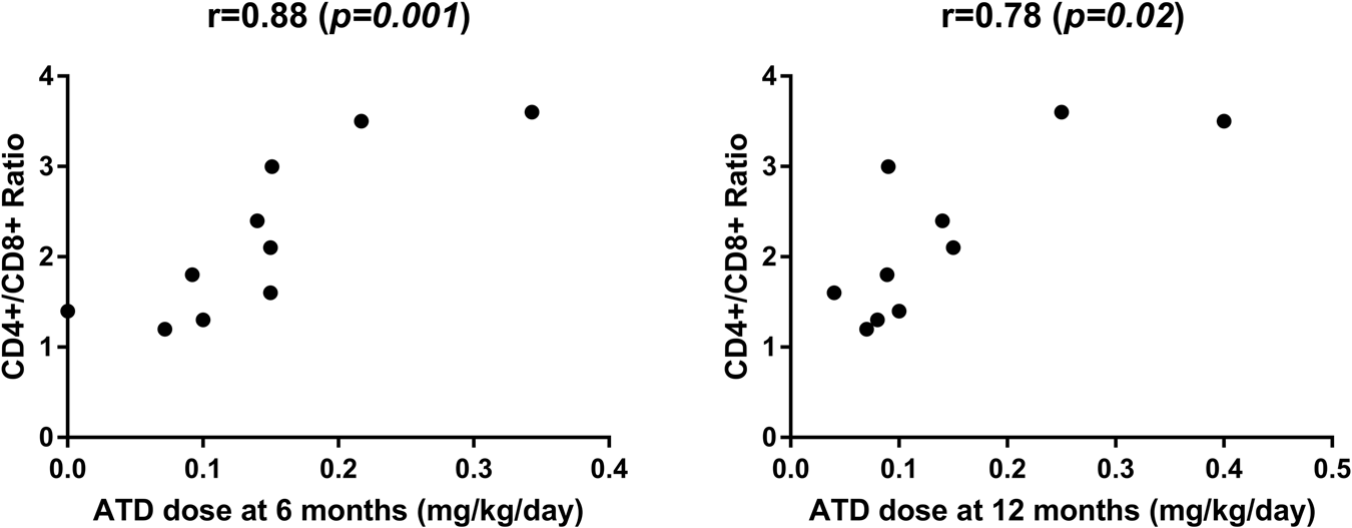
Correlation among ATD dose and CD4+/CD8+ ratio

Mean total lymphocyte count was 2.65 ± 0.7 10^9^/L (normal range 2.2–3.5), mean T cell count was 1.93 ± 0.6 10^9^/L (normal range 1.5–2.5), while mean B cell count was 0.38 ± 0.09 10^9^/L (normal range 0.44–0.77). Mean CD4+ count was 1.06 ± 0.11 10^9^/L (normal range 0.64–1.2), whereas CD8+ count was 0.65 ± 0.11 10^9^/L (normal range 0.64–0.9) and the mean ratio of CD4+/CD8+ T cell subtype was 2.19 ± 0.9 (normal range 1.2–1.7). Pearson’s correlation coefficient between CD4+/CD8+ ratio at diagnosis and fT3 levels and thyroid volume at diagnosis was 0.72 (*p* = 0.04) and 0.81 *(p* = *0.004)* respectively (Fig. 1). As shown in Fig. 2, a positive correlation coefficient was also observed between the CD4+/CD8+ ratio at diagnosis and the TRAb titre at diagnosis and after 6, 12 and 24 months (0.89, 0.89, 0.73 and 0.77; *p* = *0.02*, *p* = *0.01*, *p* = *0.03* and *0.04* respectively). The correlation coefficient of the ATD dose after 6 and 12 months with the CD4+/CD8 ratio also showed a similar course (0.88 and 0.78, *p* = *0.001* and *p* = *0.02* respectively, Fig. 3). Patients with a CD4+/CD8 + ratio above normal (6/10) had higher levels of fT3 at diagnosis (28.77 ± 2.18 vs 13.48 ± 2.19 pmol/L, *p* = *0.03*) and higher TRAb titre (28.9 ± 11.2 vs 4.88 ± 0.97, *p* = *0.01*) compared to subjects with normal ratio (4/10).

After 2 years of follow-up, 2/10 patients underwent thyroidectomy for a persistent thyrotoxic state despite the increase of ATD dose, 3/10 had remission of the disease and 6/10 are still being treated with ATD. The differences of clinical features at GD onset are represented in Table 2.

**Table 2:** Difference of clinical features at GD onset in subjects with disease remission and definitive surgical approach or long-term medical treatment

	Disease remission (*n* = 3)	Surgical approach (*n* = 2)	Long-term medical treatment (*n* = 5)	*p*
TSH mUI/l (0.6–4.84)	0.005 ± 0.001	0.005 ± 0.01	0.005 ± 0.001	0.86
FT4 pmol/L (8.6–14.9)	43.1 ± 5.92	57.8 ± 18.8	36.5 ± 4.2	0.23
FT3 pmol/L (1.87–3.99)	12.5 ± 2.5	23.7 ± 8.75	13.89 ± 2.55	0.2
TRAb UI/L (<3.5)	9.34 ± 5.4	53.8 ± 30.05	11.34 ± 3.3	**0.04**
Thyroid volume SDS	1.93 ± 0.52	5.04 ± 0.06	2.79 ± 0.58	**0.04**
Total lymphocyte count 10^9^/L (2.2–3.5)	2.07 ± 0.96	1.76 ± 0.25	2.82 ± 0.23	0.4
T cells 10^9^/L (1.5–2.5)	2.34 ± 0.4	1.23 ± 0.66	1.96 ± 0.18	0.12
CD4+ 10^9^/L (0.64–1.2)	1.3 ± 0.26	0.67 ± 0.24	1.07 ± 0.89	0.15
CD8+ 10^9^/L (0.64–0.9)	0.85 ± 0.15	0.26 ± 0.05	0.69 ± 0.15	0.14
CD4+ /CD8+ ratio (1.2–1.7)	1.46 ± 0.17	3.55 ± 0.05	2.08 ± 0.29	**0.01**
B cells 10^9^/L (0.44–0.77)	0.29 ± 0.06	0.4 ± 0.05	0.43 ± 0.03	0.11
ATD dose (mg/kg/day)	0.32 ± 0.1	0.35 ± 0.15	0.3 ± 0.54	0.78

Bold values identify statistical significance (*P* < 0.05).

CD4+/CD8+ ratio was higher in the subjects who underwent thyroidectomy with respect of subjects who had disease remission and long-term medical treatment approach (3.55 ± 0.05 vs 1.46 ± 0.17 vs 2.08 ± 0.29 respectively, *p* = *0.01*). The same trend was observed for TRAb levels (53.8 ± 30.05 vs 9.34 ± 5.4 vs 11.34 ± 3.3 UI/L respectively, *p* = *0.04*) and thyroid volume (5.04 ± 0.06 vs 1.93 ± 0.52 vs 2.79 ± 0.58 respectively, *p* = *0.04*). Scheffe, LSD and Duncan Bonferroni tests confirmed the differences in TRAb levels and thyroid volume between patients in the remission group and those who underwent definitive treatment, while the CD4+/CD8+ ratio was significantly different between the three categories of patients (remission vs definitive treatment vs long-term treatment).

The relative risk (RR) of taking a higher daily dose of ATD than maintenance dose (0.1–0.2 mg/kg/daily) and a higher CD4+/CD8+ ratio was 4 and 2.25 at 6 and 12 months from diagnosis respectively. The RR of having a thyroid volume greater than SDS > 2 and a higher CD4+/CD8+ ratio was 3.

## Discussion

Graves Disease (GD) is the most common cause of thyrotoxicosis in pediatric age; however, its management is still challenging with many questions still open on the duration of the medical approach with ATD and when definitive treatment should be considered. Currently, ATD treatment is administered for at least 3 years and can be stopped when TRAb levels have been low for several months. Longer periods of ATD may be considered if remission likelihood is low. Definitive treatment should be considered when patients remain thyrotoxic despite the use of high doses of ATD, in patients who develop serious ATD-related side effects and in subjects with relapse [3].

Most Authors report lower remission rates than adults (15–40% vs 40–85%) with patients relapsing mainly in the first 6–12 months after ATD discontinuation. The overall remission rate during ATD treatment in pediatric age is 28.8% [20]. Younger age, large goiter size, higher fT4 and fT3 levels, higher TRAb titration, and duration of ATD treatment itself, are believed to be important predictive factors [9–13]. The availability of predictive factors in GD is important as tools that can drive the pediatric endocrinologist in choosing between short-term medical treatment and subsequent definitive approach, long-term medical treatment, or the direct definitive approach. Choosing the right treatment at the right time is of primary importance in pediatric GD, considering the low rate of remission reported and the poor quality of life of children and adolescents during the thyrotoxic state.

To date, few data are available on the correlation of lymphocyte parameters and the outcome of GD in pediatric age. Chen et al. observed a higher CD4+/CD8+ ratio, lower percentages of CD3+ CD8+ T cells and higher percentages of CD3− CD19+ cells in children and adolescents with Grave’s ophthalmopathy (GO), although non independent after excluding effect of the TRAb titre [16]. In another study no correlation was observed between peripheral blood naive T lymphocyte and levels of TRAb or fT4 levels, but the increase in transient and pre-naive mature B lymphocytes was related to plasma levels of fT4 [18]. In GD, CD4+ cells significantly increase in the thyroid tissue, while the number of CD8+ cells increases to a lesser extent both in the parenchyma and lymphatic infiltrations and decreases in peripheral blood [15, 17]. The correlation between TRAb levels and B lymphocytes was confirmed, whereas no correlation has been found between TRAb levels and the percentage of lymphocyte subpopulations in thyroid tissue [16]. Given the crucial role of TRAb in the pathogenesis of GD, it is reasonable to hypothesize that the greater the increase of CD4+ and the CD4+/CD8+ ratio, the more severe the manifestation of GD, although this has not yet been confirmed as an indipendent variable.

Immunological and hormonal remission were considered as parameters of disease severity, with patients undergoing definitive treatment classified as having the most severe manifestation of GD. In our study the CD4+/CD8+ ratio was the only lymphocyte parameter significantly related to disease severity as subjects underwent thyroidectomy after 2 years of follow-up showed higher ratio than subjects with disease remission and long-term medical treatment approach.

A positive correlation was observed between CD4+/CD8+ ratio and fT3 levels and diagnosis and TRAb titre at diagnosis and after 6, 12, and 24 months. The same trend was also observed for thyroid volume and the daily dose of ATD. Subjects with a higher CD4+/CD8+ ratio also had a higher risk of having a thyroid volume >2 SDS and a higher ATD dose. Patients with a higher ratio seem to be at higher risk of developing the more severe manifestations of GD (patients undergoing definitive treatment after 2 years of follow-up or to long-term treatment approach, even if this hypothesis need to be confirmed in larger multicentric studies with greater cohorts, given the rarity of GD. We believe that this lymphocyte subset parameter should be considered as predictive for outcome, along with other factors considered, such as age, sex, fT3 and TRAb levels at diagnosis and thyroid volume. To our knowledge, this is the first study in pediatric age reporting the relation of CD4+/CD8+ ratio to disease severity. Lymphocyte subgroups should be evaluated at the time of GD diagnosis as methimazole and other ATD drugs interfere with the immune system.

The limitations of the present study lie mainly in the size of the cohort which limits the statistical strength of our data, although the rarity of GD in pediatric age must be considered.

Among the parameters of the lymphocyte population, the elevated CD4+/CD8+ ratio appears to be positively correlated with higher fT3 levels and goiter size at diagnosis, higher TRAb titre in the first 2 years, and higher ATD dose in the first year after the diagnosis. Thus, this ratio could be considered as a promising predictive tool, together with the other prognostic factors to better manage pediatric GD, even if these preliminary data need to be confirmed over a longer follow-up period and in larger cohorts.
